# Is estimated bullet trajectory a reliable predictor of severe injury? Case report of a thoraco-abdominal gunshot with a protracted trajectory managed nonoperatively

**DOI:** 10.1186/1756-0500-6-63

**Published:** 2013-02-15

**Authors:** Muhammad Sohaib Khan, Bilal Masood Khan, Sumbul Naz, Muhammad Taqi Pirzada

**Affiliations:** 1 Surgical Unit-6, Civil Hospital, Baba-e-Urdu Road, Karachi, Pakistan

## Abstract

**Background:**

Operative management of all gunshot’s traumas carries a high rate of unwarranted interventions that are known to cause serious complications. Selective nonoperative management is thus being increasingly practiced which has reduced these avoidable interventions. Physical examination and computed tomography scans are most sensitive in assessing need of laparotomy. Assessment of internal injuries on the basis of an estimated bullet trajectory is often practiced but has seldom been studied. We report a case of conservative management of a thoraco abdominal gun shot patient where an estimated bullet trajectory was indicative of serious injuries. To the best of our knowledge this is the first report of a thoraco abdominal gunshot that, despite of a protracted trajectory, had no sequelae and was thus managed nonoperatively.

**Case presentation:**

A 30 year old male patient having height of 180 cm and weight of 70 kg (Body Mass Index 21.6) presented with complaint of a penetrating injury at left side of upper torso. The patient had no symptoms or obvious bleeding and was vitally stable. On examination a 1 cm × 1 cm entry wound at the left 3rd intercostal space in the mid clavicular line was identified. The chest and abdomen were otherwise unremarkable on examination. The chest radiograph displayed clear lung fields. The abdominal radiographs displayed a bullet in the upper left quadrant of the abdomen lateral to the spine. The bullets estimated trajectory from 3rd intercostal space and its lodgment in the abdomen lateral to the spine indicated severe visceral injury. The computed tomography scan showed that the bullet was lodged postero-medially to the left kidney. All thoracic, intra peritoneal and retroperitoneal visceral structures were identified to be normal. The patient remained clinically and vitally stable, hence was managed nonoperatively being discharged after 48 h of observation.

**Conclusion:**

From this case we conclude that decision for managing gun shot patients should be based on objective clinical and diagnostic findings. We recommend further investigation of the predictability of estimated trajectory for visceral injuries and consequent operative intervention as we found it to be misleading in this case.

## Background

Operative intervention used to be the standard management of all penetrating gunshot’s traumas. Due to the high rate of these interventions being identified as unwarranted, there has been an increasing trend towards nonoperative management as many of these avoidable interventions result in serious complications. Negative laparotomies for penetrating abdominal trauma have been reported in 6% to 21.7% of cases [1,2]. Up to 41% of negative laparotomies have been associated with serious complications [3]. Nonoperative management has been greatly instrumental in decreasing rates of avoidable interventions. In a series of 1856 abdominal gunshot patients reported by Velmahos [4], rate of unnecessary laparotomies would have been 47% as opposed to 9% if 712 patients were not managed conservatively.

Physical examination is one of the most sensitive and efficient means of assessing the need of laparotomy in gun shot patients [5-7]. Computed tomography (CT) scan is the most sensitive diagnostic imaging modality for visualizing visceral injuries in gunshot patients [8,9].

Trauma surgeons often evaluate internal injuries on the basis of an estimated bullet trajectory but the predictability of this means has seldom been studied [10]. An estimated bullet trajectory was established to be unreliable by Demetriades [7] as out of the 224 patients that were estimated to have severe intra peritoneal injuries, only 75% required operative interventions. Razzaq [10] quantified a correlation of only 31% between the expected injuries from the entry wounds and observed intra peritoneal injuries during laparotomy. We report a case of a thoraco abdominal gun shot patient where an estimated bullet trajectory was indicative of serious injuries but due to the hemodynamic and clinical stability, the patient was successfully managed conservatively. To the best of our knowledge this is the first report of a single gunshot penetration of thoraco abdominal cavity, which despite of a protracted trajectory, had no sequelae and was thus managed nonoperatively.

## Case presentation

A 30 year old male patient having height of 180 cm and weight of 70 kg (Body Mass Index 21.6) presented to the emergency department of Civil Hospital Karachi with the complaint of a penetrating injury at the left side of upper torso. The patient was lying comfortably with no symptoms of respiratory distress or any obvious active bleeding. Vitally the patient was stable. There was slight blood staining of the upper garment with an obvious breach.

On examination a 1 cm × 1 cm entry wound at the left 3rd intercostal space in the mid clavicular line was identified. The chest and abdomen were otherwise unremarkable. Normal vesicular breathing was appreciated on chest auscultation. On palpation the abdomen was soft with no peritoneal signs.

**Figure 1:**
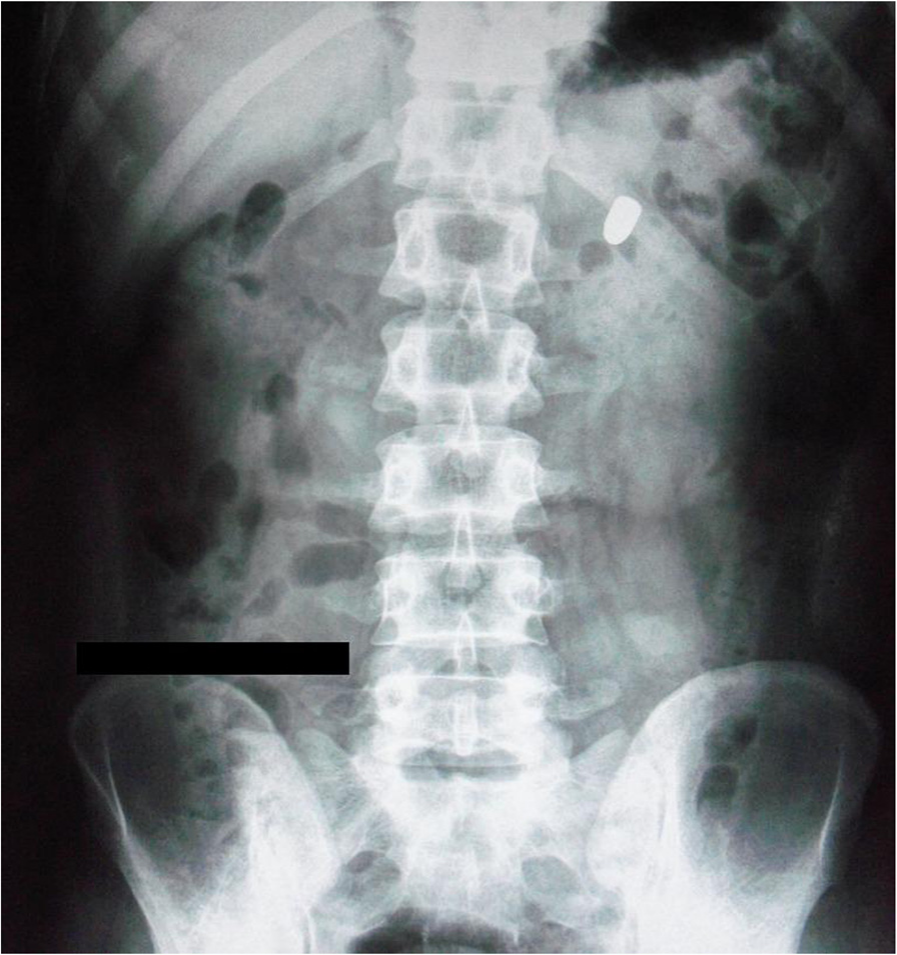
**Erect abdominal radiograph.** Radiograph showing bullet in the upper left quadrant of the abdomen.

**Figure 2:**
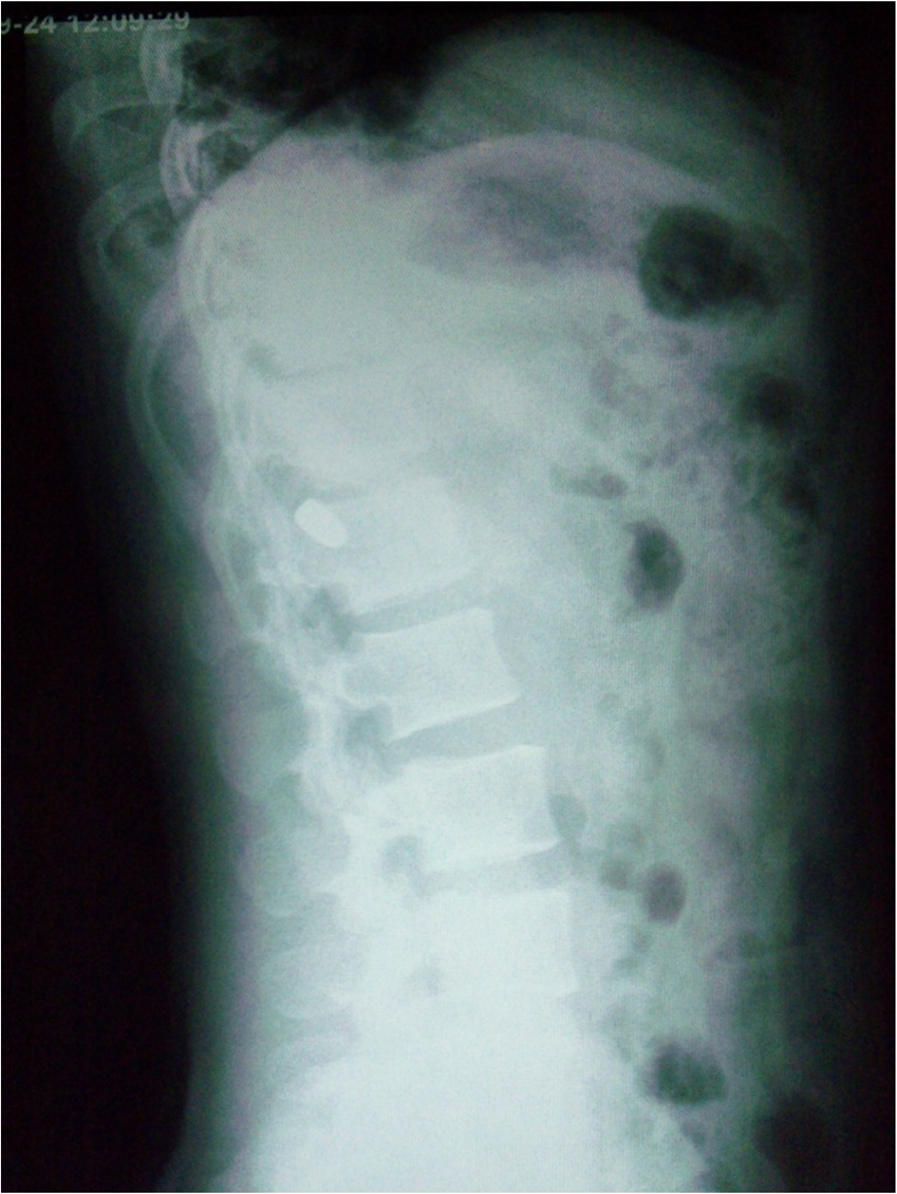
**Lateral abdominal radiograph of the patient.** Radiograph showing bullet to be lateral to the spine.

Chest radiograph demonstrated clear lung fields with no indication of injury. The abdominal radiographs (Figures 1 and 2) displayed a bullet in the upper left quadrant of the abdomen lateral to spine. The radiographs were otherwise unremarkable for any other abnormality.

With this evidence of the bullets entry at 3rd intercostal space laterally to the mid clavicular line and its lodgment in the abdomen just lateral to the spine, the bullets trajectory was estimated to have encountered the left lung, left dome of the diaphragm, stomach, left lobe of the liver, splenic vessels, body of the pancreas and the left kidney. Despite this trajectory, the patient was clinically stable with no signs of either respiratory distress or any other visceral injury. An urgent CT scan was advised to identify any serious damage as was being predicted on the basis of the estimated trajectory.

**Figure 3:**
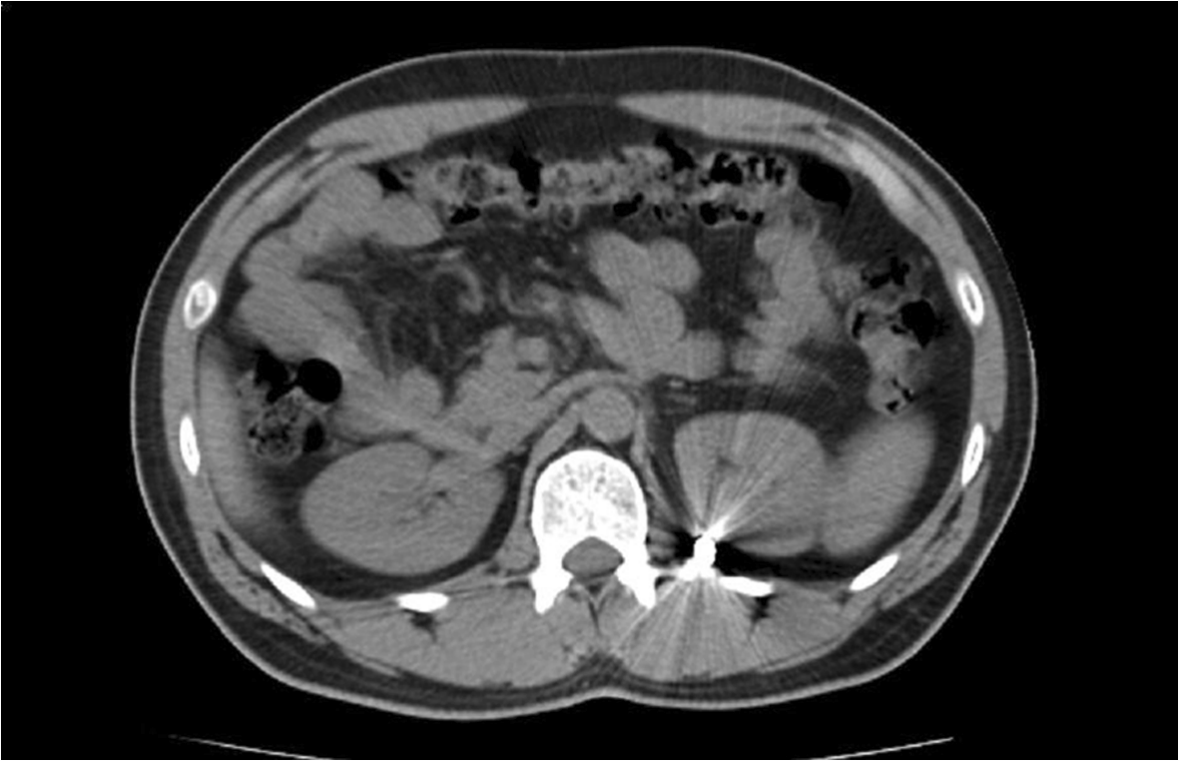
**Transverse view of CT scan.** This view shows the bullet to be lodged postero medially to the left kidney.

The CT scan (Figure 3) showed that the bullet was lying postero medially to the left kidney just lateral to the spine. All thoracic, intra peritoneal and retroperitoneal visceral structures were identified to be normal with no injury.

As the patient remained clinically and vitally stable with there being clear evidence of all visceral structures being unharmed from the bullet, the patient was admitted for nonoperative management and observation. Patients’ hemoglobin (Hb) concentration and other baseline investigations were performed on admission and subsequently after every 24 h. There was no drop in the Hb from the initial measurement of 12.5 mg/dl. Serial clinical examinations were performed that remained negative for any peritoneal signs, visceral damage or respiratory distress. The patient was therefore discharged after 48 h of observation.

## Discussion

Penetrating gunshots, due to their destructive nature for all structures in their path, are considered to pose an imminent threat to the victims’ life unless proven otherwise. For this reason, early recognition of the damage and timely intervention is of utmost importance in saving the patients’ life. High degree of caution thus observed by trauma surgeons, though necessary for appropriate management of gunshot victims, has often resulted in operative interventions which were avoidable. Negative laparotomies for penetrating abdominal trauma have been reported in 6% to 21.7% of cases [1,2]. These negative laparotomies in up to 41% of cases have been reported by Renz [3] to be the cause of serious complications that include atelectasis, postoperative hypertension, pleural effusion, pneumothorax, prolonged ileus, pneumonia, surgical wound infection and small bowel obstruction. These result in greatly increased lengths of hospital stay ranging from 5.3 days when there was no associated injury to more then 11 days when associated with injuries [11].

To avoid these negative laparotomies and their associated morbidities, in 1974 Nance [12] advocated the use of careful conservative management for abdominal gun shot patients. Since then, the increasing trend of nonoperative management of selected patients has been greatly instrumental in decreasing the rate of negative laparotomies. Velmahos [4] and colleagues published a series of 1856 abdominal gunshot patients that presented over an 8 year period of which 712 were managed conservatively. Rate of negative laparotomies was 9% which would have been 47% if patients were not managed nonoperatively. Selective nonoperative management enabled the trauma team in this series to save a calculated total of 3560 hospital days and $9,555,752 in hospital charges. Conservative management is now being practiced where even liver injuries have been diagnosed. Navsaria [13] and colleagues conducted a prospective study on patients with diagnosed liver injuries in abdominal and thoraco-abdominal gunshots. Out of the 63 patients initially managed nonoperatively, only 5 patients required delayed laparotomy yielding a 92% rate of successful nonoperative management. This lead the authors conclude that nonoperative management can be safely and effectively practiced for selected patients with liver gunshot injuries unrelated to its severity.

Serial clinical examination and CT scan have been found to be most sensitive and reliable in defining the need for operative intervention in gunshot patients [14]. The sensitivity of clinical examination in anterior abdominal gunshots has been reported to be 97% [7] while for trans pelvic gun shots and for gun shots to the back, sensitivity its reported to be 100% [5,6]. CT scan is the most sensitive diagnostic imaging modality for visualizing visceral injuries in gunshot patients [8,9]. It was because of the clinical and vital stability of our patient with evidence of no visceral injury from the CT scan, that we in this case were able to successfully manage a thoraco abdominal gunshot patient nonoperatively.

Surface entry and exit wounds of gunshots with an estimated trajectory are often taken to be predictors of visceral injury but have been seldom studied [10,11]. Such estimation was found to be highly unreliable by Razzak [10] who using the medical records of patients quantified a correlation between estimated trajectory and observed internal injuries. A mean correlation of 31% was quantified being as low as 9% for mobile viscera like small intestine. Similarly, Demetraides [7] also established the unreliability of estimated trajectory as out of the 224 patients with peritoneal penetration, only 75% had severe injuries requiring operative intervention.

We also report a case where an estimated trajectory from the bullets entry at the 3rd intercostals space to its lodgment postero medially to the left kidney indicated multiple visceral injuries that include left lung, left dome of the diaphragm, stomach, left lobe of the liver, splenic vessels, body of the pancreas and the left kidney. However all of these organs remained unharmed despite this estimated trajectory allowing the patient to stay clinically and vitally stable thus preventing any intervention from the trauma team.

## Conclusion

From this case and with the above review of literature, we conclude that decision for managing gun shot patients should be based on objective clinical and diagnostic findings. We recommend further investigation on the predictability of estimated trajectory as we found it to be misleading in the diagnosis of visceral injuries and consequent need of operative intervention, even in a patient with thoraco abdominal gunshot with a protracted trajectory as reported in this case.

## Consent

Written informed consent was obtained from the patient for publication of this case report and accompanying images. A copy of the written consent is available for review by the Editor-in-Chief of this journal.

## Competing interests

The authors declare that they have no competing interests’.

## Authors’ contributions

MS being the lead intern in management of the case identified it for reporting, assessed its importance and significance though literature search and wrote the manuscript. BM was at forefront of the nonoperative management of the patient and contributed towards the writing of manuscript. SN was the lead resident who monitored the patients’ condition from admission till discharge; she also assembled data of the case. MT supervised the management of the patient as well as writing of the manuscript. The final manuscript was read and approved by all the authors.
